# Native shrubs senesce earlier and faster than non-native shrubs in a temperate deciduous woodland in south-eastern Wisconsin, USA

**DOI:** 10.1007/s00484-026-03252-y

**Published:** 2026-06-22

**Authors:** Alison Donnelly, Elisabet M. Jatmiko

**Affiliations:** Department of Geography, University of Wisconsin-Milwaukee, Milwaukee, WI 53201 USA

**Keywords:** Autumn phenology, Native and non-native temperate deciduous shrubs, Chlorophyll degradation, SPAD chlorophyll meter

## Abstract

Shrubs, both native and non-native, play a pivotal role in temperate deciduous forest dynamics by providing food and habitat for a range of organisms, contributing to nutrient and carbon cycling and particularly in the case of non-native shrubs, extending the growing season. Phenological patterns in shrubs differ from trees, including earlier leaf-out and delayed leaf senescence, which prolongs the photosynthetic active period and enhances carbon uptake. Despite their ecological significance shrubs remain understudied. The objective of this research was to quantify variations in autumn leaf senescence timing and rates between native and non-native shrubs using both in situ observations and chlorophyll meter (SPAD) readings. Phenological observations and SPAD values were recorded twice weekly throughout the autumn season (2018-2024) on 5 native and 4 non-native shrub species in a small (4.5 ha) temperate deciduous woodland fragment on the University of Wisconsin-Milwaukee campus. Results show that non-native shrubs retain green leaves roughly 3-weeks longer than native species. Chlorophyll meter readings support this trend, showing prolonged chlorophyll retention and a more abrupt decline in leaf chlorophyll content in non-native shrubs. These findings improve our understanding of how shrubs shape understory forest phenology by quantifying how the pattern of senescence differs between native and non-native shrubs which in turn will have implications for the timing of understory light availability and supply of organic matter for nutrient recycling. In addition, these findings strengthen our knowledge of the ecological role of native and non-native species in temperate deciduous forest understories.

**Supplementary Information:**

The online version contains supplementary material available at 10.1007/s00484-026-03252-y.

## Introduction

Despite the dominance of trees in temperate deciduous forest communities, understory shrubs play a pivotal role in overall ecosystem functioning. Shrubs provide both habitat and food for various wildlife species, including birds, insects, and small mammals. Shrubs also play an influential role in forest phenology – particularly at the extremes of the growing season, they increase species composition and contribute to a range of ecosystem processes, yet they remain under-studied (reviewed in Donnelly and Yu 2019; Campioli et al. 2025). Phenological patterns in shrubs exhibit earlier leaf-out in spring and delayed leaf senescence in autumn, compared to trees, thus contributing to an extension of the overall growing season. However, when non-native shrubs are present the natural pattern is disrupted (Fridley 2012; Donnelly et al. 2024a, 2024b). A recent study revealed that, on average, the shrub community in a temperate deciduous forest began to senesce at the same time as the trees but the non-native shrubs stayed greener longer and retained their leaves for nearly a month after the trees and native shrubs became leafless (Donnelly et al. 2024b). This, in turn, has implications for the timing of light availability on the forest floor, nutrient dynamics and carbon assimilation.

The primary function of leaf senescence is the recovery of nutrients prior to leaf abscission and the preparation of plants for winter. Nitrogen is generally remobilized and stored in woody tissue during dormancy and subsequently flows to newly formed tissue when the growing season resumes in spring (Estiarte and Peñuelas 2015). In recent decades, the timing of leaf senescence in deciduous trees has become delayed by 1-2.5 days per decade as a result of climate change (e.g. Peñuelas et al. 2002 (European Mediterranean); Menzel et al. 2006 (Europe); Matsumoto et al. 2003 (Japan). Delayed leaf senescence may expose leaves to frost damage which may prevent nutrient resorption thus limiting nutrient reserves for leaf growth the following spring (Estiarte and Peñuelas 2015; Schreiber et al. 2013). A recent review (Campioli et al. 2024) concluded that more research on the phenological sensitivity of over- and understory forest species was required to comprehensively address the future impact of climate change on forest phenology.

In autumn, leaf senescence and leaf fall of shrubs influence nutrient dynamics by contributing leaf litter to the forest floor for decomposition and nutrient cycling which in turn influence soil fertility. Therefore, understanding the timing and duration of autumn phenology is an important contributing factor to biogeochemical cycles (Keenan et al. 2014). In a meta-analysis of a range of vegetation types including deciduous and evergreen trees and shrubs, primarily from the USA and Europe results suggested that roughly half the nitrogen (and phosphorous) contained in mature leaves (deciduous/evergreen shrubs and trees) was resorbed during the senescence process and used for further plant growth thus reducing dependence on nutrient uptake in spring (Aerts 1996). A more recent review (Vergutz et al. 2012), examining woody and non-woody species from many global locations, suggested that on average 62% of leaf nitrogen is resorbed before abscission with shrubs having higher nitrogen resorption efficiencies than trees (Sophia et al. 2024). While yet another study albeit on one species (*Populus tremula*) reported nutrient resorption efficiencies of up to 90% were possible (Keskitalo et al. 2005) however, interannual variation in resorption rates was considerable (Killingbeck 1996). The remaining nutrients are circulated through the ecosystem following leaf fall and subsequent decomposition. Therefore, leaf senescence patterns play an important role in nutrient cycling and thus the productivity and health of forests (Aerts 1996; Vergutz et al. 2012).

Leaf senescence can be tracked over the autumn season at the plant level by direct in situ phenological observations and at the leaf level by using a hand-held chlorophyll meter, such as the Minolta SPAD-502 (Soil Plant Analysis Development). This non-destructive portable device compares absorbance in the red (~ 650 nm, peak Chl absorbance) and near-infrared (~ 940 nm non-Chl absorbance) parts of the spectrum with the difference being proportional to foliar chlorophyll content (Markwell et al. 1995). This method is widely used in agriculture but underutilized in forest science (Coste et al. 2010; Morley et al. 2020; Panici et al. 2024). A large number of readings can be obtained once the relationship between the meter readings and actual leaf chlorophyll content has been established in the laboratory. Species-specific calibration equations have been established for a range of native (*Ribes americanum* Mill., wild currant; *Prunus virginiana* L., chokecherry; *Viburnum lentago* L., nannyberry; *Cornus alternifolia* L., pagoda dogwood) and non-native (*Lonicera morrowii* A. Gray, honeysuckle; *Rhamnus cathartica* L., common buckthorn; *Ligustrum vulgare* L., European privet) temperate deciduous shrub species in midwestern USA (Donnelly et al. 2020). The authors concluded that non-native shrubs tended to have higher leaf chlorophyll content than native species at that particular site.

Since temperate deciduous shrubs and the autumn season are both understudied yet pivotal to a healthy forest community the objectives of this study were to explore interannual differences in (i) the timing and duration of senescence at the whole plant level using in situ phenological observations and (ii) the pattern of leaf-level chlorophyll degradation, between native and non-native shrubs in a temperate deciduous woodland. This is the first study of its kind to use a Minolta SPAD-502 chlorophyll meter throughout the senescence period on multiple shrub species across multiple years to quantify patterns of chlorophyll degradation. As mentioned above, a previous study (Donnelly et al. 2020) established calibration equations for the relationship between SPAD values and leaf chlorophyll content for most of the shrubs in the current study. Since chlorophyll degradation is closely linked to nutrient resorption (Keskitalo et a., 2005), particularly nitrogen, understanding these patterns may help assess if differences exist between native and non-native shrubs in their potential to recover nutrients before leaf fall. Furthermore, understanding shrub phenology and senescence patterns may help inform non-native species management strategies and ensure successful conservation efforts.

## Materials and methods

### Site and shrub characteristics

Data for this study were collected during the autumn seasons of 2018 to 2024, in Downer Woods (43°4’52”N, 87°52’51”W); a small (4.5 ha) urban woodland fragment on the University of Wisconsin-Milwaukee campus (southeastern Wisconsin, USA). The annual average temperature at this site is roughly 10 °C and annual average precipitation is 900 mm. The site comprises temperate deciduous trees and shrubs (native and non-native) which are regularly observed in spring and autumn and phenology recorded.

Data for 2021 are missing due to the observer being on sabbatical and data for the non-native *Viburnum opulus* (Highbush cranberry) are missing for 2023 as a result of an infestation of the Viburnum leaf beetle (*Pyrrhalta viburni*) which defoliated these plants. Due to the susceptibility of shrubs to attack by pests, pathogens and accidental death it was not always possible to monitor the same plants for the duration of the study. Details of these incidents (e.g. attack by powdery mildew or dead trees falling on shrubs) are available in Donnelly et al. (2024a). In these cases, nearby healthy individuals, of similar size and canopy structure were selected, to replace the diseased or dead plants. Furthermore, some species (see later for details) were added to the monitoring campaign after it began. Even though we did not monitor the same individuals or the same number of individuals of each species each year, we are confident that the data collected remains representative of the overall shrub phenology at the site.

### In situ phenological observations

Throughout the autumn seasons (2018–2024) in-situ phenological observations were recorded on native and non-native temperate deciduous shrubs two times per week (Table 1). On each occasion, each individual plant was observed and the percentage of leaves showing signs of autumn senescence was recorded. Senescence progression was recorded using percentage color. The first stage was < 10% (meaning that less than 10% of the leaves on an individual plant showed signs of senescence (color). Subsequent stages were 10–20%, 20–30%, 30–40%, 40–50%, 50–60%, 60–70%, 70–80%, 80–90% and > 90%. All in situ observation records are available in the data repository PANGAEA (Donnelly 2024b, 2025).

**Table 1:** List of temperate deciduous shrub species monitored at Downer Woods, Wisconsin, USA (2018–2024). The number (N) of individual plants for some species varied across years due to insect damage, disease and death. N(yr) is the number of years each species was observed

Plant group		Species	*N*	*N*(yr)
Shrub species	Native	*Prunus virginiana* (Chokecherry)	4–5	6
		*Cornus alternifolia* (Pagoda dogwood)	2–3	6
		*Viburnum lentago* (Nannyberry)	5	6
		*Ribes americanum* (American Wild currant)	5	5
		*Euonymus atropurpureus* (Eastern wahoo) added in 2018	2	4
	Non-native	*Rhamnus cathartica* (Buckthorn). Introduced from Europe as an ornamental.	5	6
		*Lonicera morrowii* (Honeysuckle). Introduced from Asia as an ornamental.	1–3	6
		*Ligustrum vulgare* (European Privet). Introduced from Europe as an ornamental.	3	6
		*Viburnum opulus* (European Highbush cranberry). Introduced from Europe as an ornamental.	7–9	5

### SPAD meter data collection and leaf chlorophyll determination

Twice weekly, from August – December (2018 to 2024), SPAD (Soil Plant Analysis Development, Konica-Minolta, Japan, SPAD-502) values were recorded on five leaves of each individual plant (Table 1). SPAD values were recorded on the same days as in situ observations were made. All SPAD values are available in the data repository PANGAEA (Donnelly 2024a).

Towards the end of the senescence period, leaves began to exhibit greater color variation i.e. the degree of ‘coloring’ was not even across the surface of the leaf. This resulted in some parts of the leaf being green while others were completely senesced. Even though care was taken to obtain SPAD values on the same place on the leaf each time, on occasion SPAD values increased when a decrease was expected. For example, in 2024 SPAD values for one of the Honeysuckle plants increased by 10 units between DOY 298 (17.65) and DOY 302 (27.26) and subsequently decreased to 7.5 by DOY 309. In this case we felt justified in removing the data for DOY 302 from further analysis. Table S1 presents a list of 31 instances in which data were omitted from further analysis. It is interesting to note that a very small number of data points were removed, and they were usually within the last 3 days in the time series. SPAD data for Wild currant (*Ribes americanum*) in 2024 were omitted from further analysis because very few leaves remained on these plants in autumn, likely due to it being the first species to leaf-out in spring in response to unusually warm temperatures (Donnelly et al. 2026).

Due to the interspecific variation in optical properties of leaves it is not advisable to compare SPAD values across species. Conversion of SPAD values to total leaf chlorophyll content (mg cm^− 2^) was made using the species specific calibration equations determined previously (Donnelly et al. 2020). A calibration equation was not available for Eastern wahoo (*Euonymus atropurpureus*) because this species of native shrub was added to the monitoring program after the 2020 study. In this case we used the average calibration for all native shrubs (Donnelly et al. 2020). A small number (5) of negative values were returned when the SPAD value was particularly low and these have also been removed (Table S2).

### Temperature data

Temperature (°F) was recorded continuously every 10 min by a HOBO sensor within the study area. The data were then aggregated to daily average temperatures (Estrella and Menzel 2006; Menzel et al. 2006) and converted to °C. However, temperature data from 12 September to 8 November 2019 were missing due to an instrument malfunction. In order to maintain a continuous temperature record, we downloaded daily high/low temperatures (°F) for 2019 for General Mitchell Airport (located 20 km from study site) from the Wisconsin State Climatology Office (https://climatology.nelson.wisc.edu/first-order-station-climate-data/milwaukee-climate/historical-temperatures/). From these data, we calculated daily average temperatures and converted them to °C. We compared the study site data for daily average temperature (6.98 °C) to that of General Mitchell Airport (8.40 °C excluding the time period 12 September to 8 November, average daily temperature at the airport for the entire year was 8.91 °C). Given the modest difference of 1.43 °C, we substituted the airport data to fill the missing period from the study site. We recognize that this is not ideal but necessary to maintain a continuous time series.

Daily mean temperatures (°C; from DOY 220) were smoothed using a 5-day moving average. For each year, we identified two dates, (i) the first DOY when the smoothed temperature remained below 10 °C for at least three consecutive days and (ii) the first DOY when the temperature remained below 5 °C for three consecutive days. These DOYs were taken to represent the onset of cooler conditions. These analyses were conducted in Python 3.10 within Google Colab using *pandas* and *matplotlib* libraries.

### Statistical analysis

To identify the onset of autumn senescence, we modeled seasonal chlorophyll dynamics using piecewise linear regression which has the ability to capture non-linear seasonal trends. The Durban-Watson statistic was used to assess residual independence for each species-year combination (Table S3). Values close to 2 indicates no autocorrelation, values < 2 indicate positive autocorrelation and values > 2 negative autocorrelation. Most models had Durbin–Watson values between 1.5 and 2.5, indicating generally low autocorrelation, with a small number of models showing mild deviations in both directions. For each species–year combination, total chlorophyll content (mg/cm^2^) was regressed against day of year (DOY). A breakpoint was identified at the first inflection point in the trend which represented the start of a rapid decline in leaf chlorophyll content. Slopes and breakpoint positions were estimated with the Python package pwlf (v2.5.2). Vertical dashed lines in the figures indicate the breakpoint for each fit, while red trend lines represent the fitted piecewise model segments (Figs. S1 & S2). From these fits, we calculated the breakpoint DOY (start of chlorophyll loss), the number of days between the breakpoint and the last observation DOY (duration of chlorophyll loss), and the slope of the declining segment (rate of chlorophyll loss). We present the native and non-native summaries as boxplots and used univariate analysis of variance to compare the three model parameters across years. We determined that fitting piecewise regression to the averaged (native or non-native group) curves obscures variability among species. Since the breakpoint is derived from the curve shape averaging across species alters the trajectory of the curve in a non-linear manner and may shift the apparent breakpoint relative to the values obtained from individual plants. Therefore, we fitted piecewise regressions to individual species and then compared breakpoints statistically between vegetation groups.

Significant differences in the mean timing of the start of (i) in situ senescence and (ii) chlorophyll decline and the duration of these events, across years and between native and non-native shrubs were determined using univariate ANOVA. Linear regression was used to determine the relationship between the start (DOY) and duration (days) of in situ senescence and leaf chlorophyll decline, and three temperature variables i.e. average August temperature and start of autumn cooling period thresholds of 10 °C and 5 °C. All statistical tests were carried out using IBM SPSS version 30.0.

## Results

### Timing and duration of autumn senescence and chlorophyll decline (2018–2024)

**Fig. 1:**
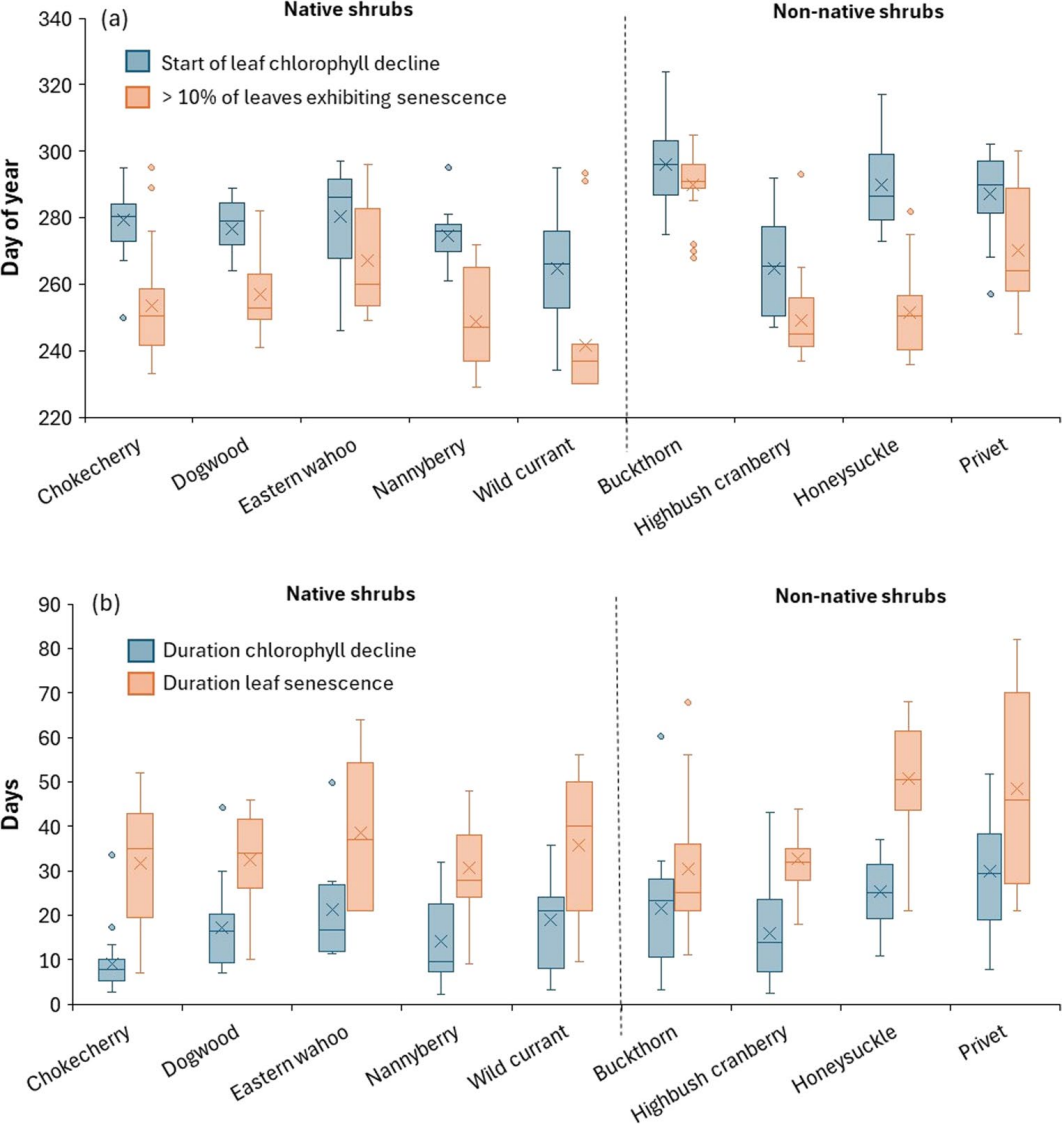
(**a**) Average start (DOY) of leaf chlorophyll decline (red boxes) as determined by piecewise linear regression inflection point and in situ observed DOY on which > 10% of leaves exhibited senescence (blue boxes) and (**b**) duration (days) of chlorophyll decline and leaf senescence for a range of 5 native temperate deciduous shrub species Chokecherry, Dogwood, Eastern wahoo, Nannyberry and Wild currant and 4 non-native shrub species Buckthorn, Highbush cranberry, Honeysuckle and Privet for 2018, 2019, 2020, 2022, 2023 and 2024

On average native shrubs began exhibiting visual signs of autumn senescence (< 10% leaves colored) on DOY 253 ± 2.5 occurring 13 days earlier (*p* < 0.001) than in non-native species (Fig. 1a). The decline in leaf chlorophyll content began approximately 21 days after the onset of visual signs of senescence. Leaf chlorophyll content (mg/cm^2^) also started to decline significantly earlier (*p* < 0.001) in native shrubs (DOY 274 ± 1.6) than in non-native shrubs (DOY 285 ± 1.6). The duration of in situ senescence (33 ± 1.7 d; *p* < 0.015) and chlorophyll decline (15 ± 1.1 d; *p* < 0.001) was also significantly shorter in native shrubs than non-natives by approximately 6 and 7 days respectively (Fig. 1b).

Across years, the onset of senescence in native shrubs started before non-native shrubs by between 1 and 20 days. These differences were statistically significant in 2020 and marginally significant in 2018 (*p* < 0.058) and 2022 (*p* < 0.059) (Table 2). A similar but stronger pattern emerged for leaf chlorophyll decline beginning 3–15 days earlier in native shrubs than non-native shrubs in most years (Table 2). The duration of both senescence and chlorophyll decline tended to be shorter in native species by 1–15 days compared to non-native shrubs but the differences were not always statistically significant (Table 3).

**Table 2:** Pairwise comparison of the timing of in situ start of senescence (DOY average ± SE) and start of chlorophyll decline (DOY average ± SE) (2018–2024). Nat; average of all native shrubs (*N* = 93), NonNat; average of all non-native shrubs (*N* = 96). P value indicates statistical significance

Year	In situ start of senescence (DOY)			Start of leaf chlorophyll decline (DOY)		
	Nat	NonNat	*P* value	Nat	NonNat	*P* value
2018	240 ± 5.6	255 ± 5.3	0.058	272 ± 3.9	287 ± 3.8	0.007
2019	243 ± 5.6	253 ± 5.2	0.182	272 ± 3.8	284 ± 3.4	0.015
2020	257 ± 5.8	277 ± 5.9	0.019	276 ± 4.2	287 ± 4.1	0.081
2022	251 ± 6.0	267 ± 6.4	0.059	277 ± 4.1	280 ± 4.1	0.038
2023	266 ± 7.0	267 ± 7.0	0.818	278 ± 4.1	289 ± 4.5	0.076
2024	260 ± 7.0	271 ± 5.6	0.190	275 ± 3.9	283 ± 3.9	0.183

**Table 3:** Pairwise comparison of the duration of in situ senescence (days average ± SE) and chlorophyll decline (days average ± SE) (2018–2024). Nat; average of all native shrubs (*N* = 93), NonNat; average of all non-native shrubs (*N* = 96). P value indicates statistical significance

Year	In situ duration of senescence (Days)			Duration of leaf chlorophyll decline (Days)		
	Nat	NonNat	*P* value	Nat	NonNat	*P* value
2018	30 ± 3.6	45 ± 5.3	0.003	19 ± 2.8	19 ± 2.7	0.967
2019	37 ± 3.6	50 ± 5.2	0.012	14 ± 2.8	25 ± 2.4	0.003
2020	31 ± 3.7	31 ± 5.9	0.993	17 ± 3.1	24 ± 2.8	0.107
2022	33 ± 3.9	37 ± 6.4	0.544	16 ± 2.8	23 ± 2.8	0.078
2023	33 ± 4.5	32 ± 7.0	0.865	17 ± 2.9	19 ± 3.2	0.747
2024	31 ± 4.3	30 ± 5.6	0.829	10 ± 2.8	23 ± 3.1	0.002

### Variation in the pattern and rate of leaf chlorophyll decline between native and non-native shrubs (2018–2024)

**Fig. 2:**
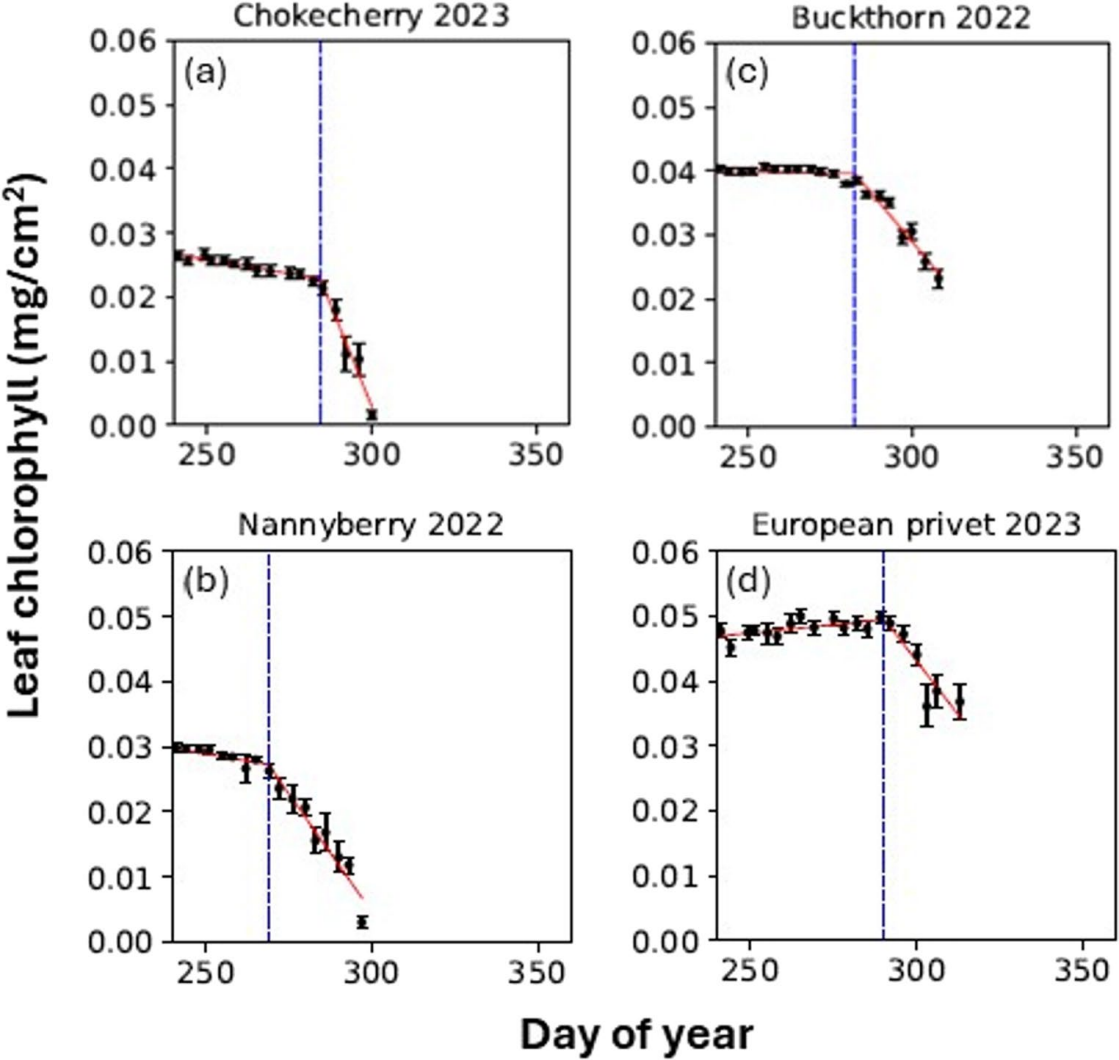
Piecewise linear regression for trends (day of year) in autumn leaf chlorophyll content (mg/cm^2^) for 2 sample native (**a**) Chokecherry and (**b**) Nannyberry and 2 non-native shrub species (**c**) Buckthorn and (**d**) European privet, for two sample years. This figure illustrates how the piecewise regression was performed, the trend in chlorophyll decline, variation between native and non-native shrubs and annual variation. Data for each species and year are available in supplemental Figs. S1 and S2. Black data points represent the average of 5 leaves ± standard error. The red trend lines represent the fitted piecewise model segments and the blue dashed line the breakpoint for each fit

As leaf chlorophyll degraded both native and non-native shrub species exhibited increasing variability in chlorophyll content (mg/cm^2^) as indicated by an increase in the length of the error bars as the season progressed (Fig. 2; Figs. S1 & S2). Leaf chlorophyll content rarely reached zero because leaves tended to drop before complete pigment loss.

**Fig. 3:**
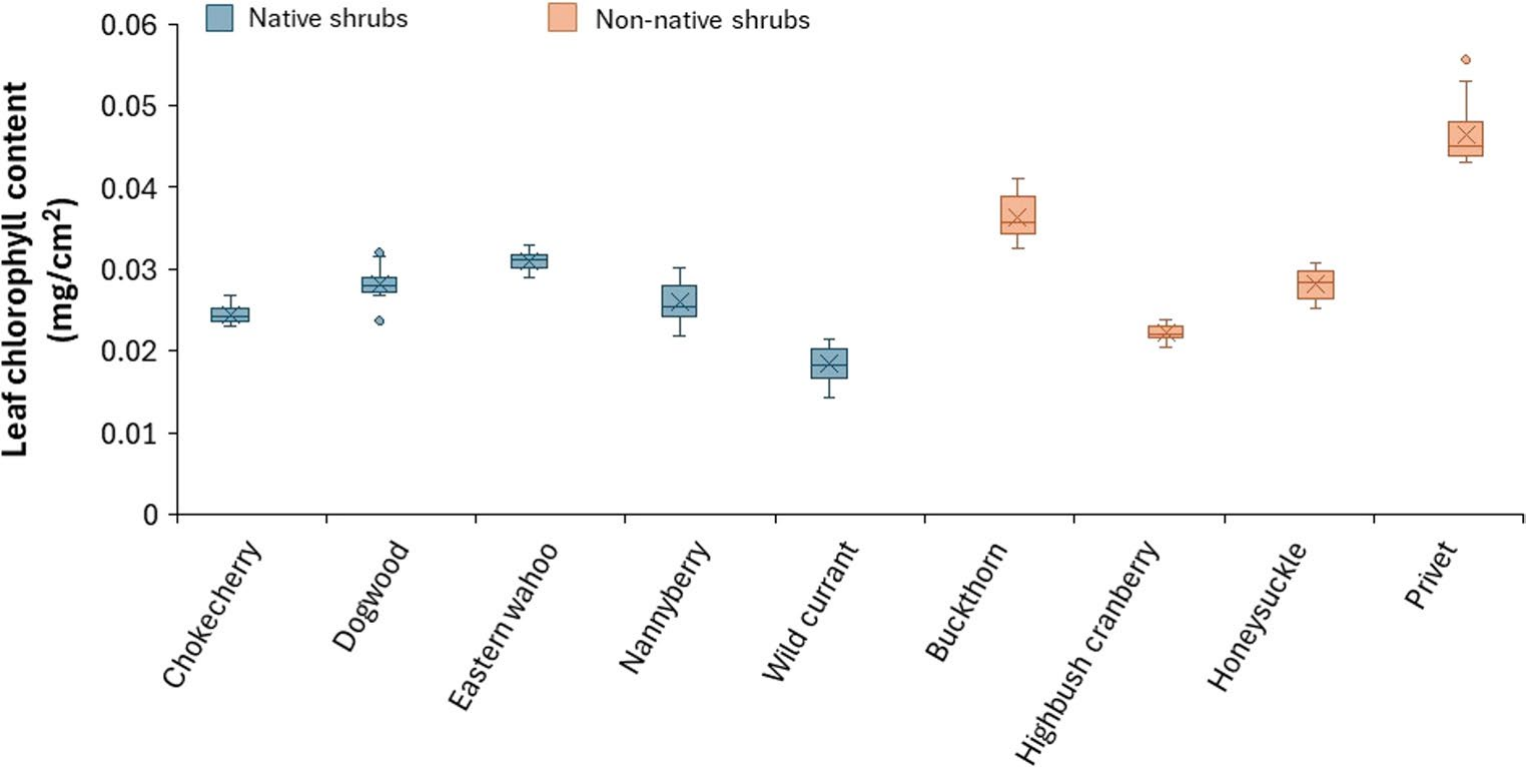
Average (2018, 2019, 2020, 2022, 2023 and 2024) leaf chlorophyll content (mg/cm^2^) to start of chlorophyll decline (DOY) for a range of 5 native temperate deciduous shrub species Chokecherry, Dogwood, Eastern wahoo, Nannyberry and Wild currant and 4 non-native shrub species Buckthorn, Highbush cranberry, Honeysuckle and Privet

The average leaf chlorophyll content (mg/cm^2^) up to the inflection point (DOY) was calculated for each species and year (Figs. S1 & S2). When averaged across years native shrubs exhibited a significantly lower leaf chlorophyll content (0.024 mg/cm^2^) than non-native shrubs by approximately 0.01 mg/cm^2^ (*p* < 0.001; Fig. 3). This pattern was consistent across all years (Table 4).

**Table 4:** Pairwise comparison of the average leaf chlorophyll content (mg/cm^2^ average ± SE) prior to chlorophyll decline (DOY) in native (Nat, *N* = 93) and non-native (NonNat, *N* = 89) shrubs (2018–2024). P value indicates statistical significance

Year	Average leaf chlorophyll content (mg/cm^2^)		
	Nat	NonNat	*P* value
2018	0.23 ± 0.001	0.32 ± 0.002	< 0.001
2019	0.24 ± 0.001	0.30 ± 0.002	< 0.015
2020	0.24 ± 0.001	0.34 ± 0.002	< 0.001
2022	0.25 ± 0.001	0.35 ± 0.002	< 0.001
2023	0.24 ± 0.001	0.37 ± 0.002	< 0.001
2024	0.26 ± 0.001	0.36 ± 0.002	< 0.001

**Fig. 4:**
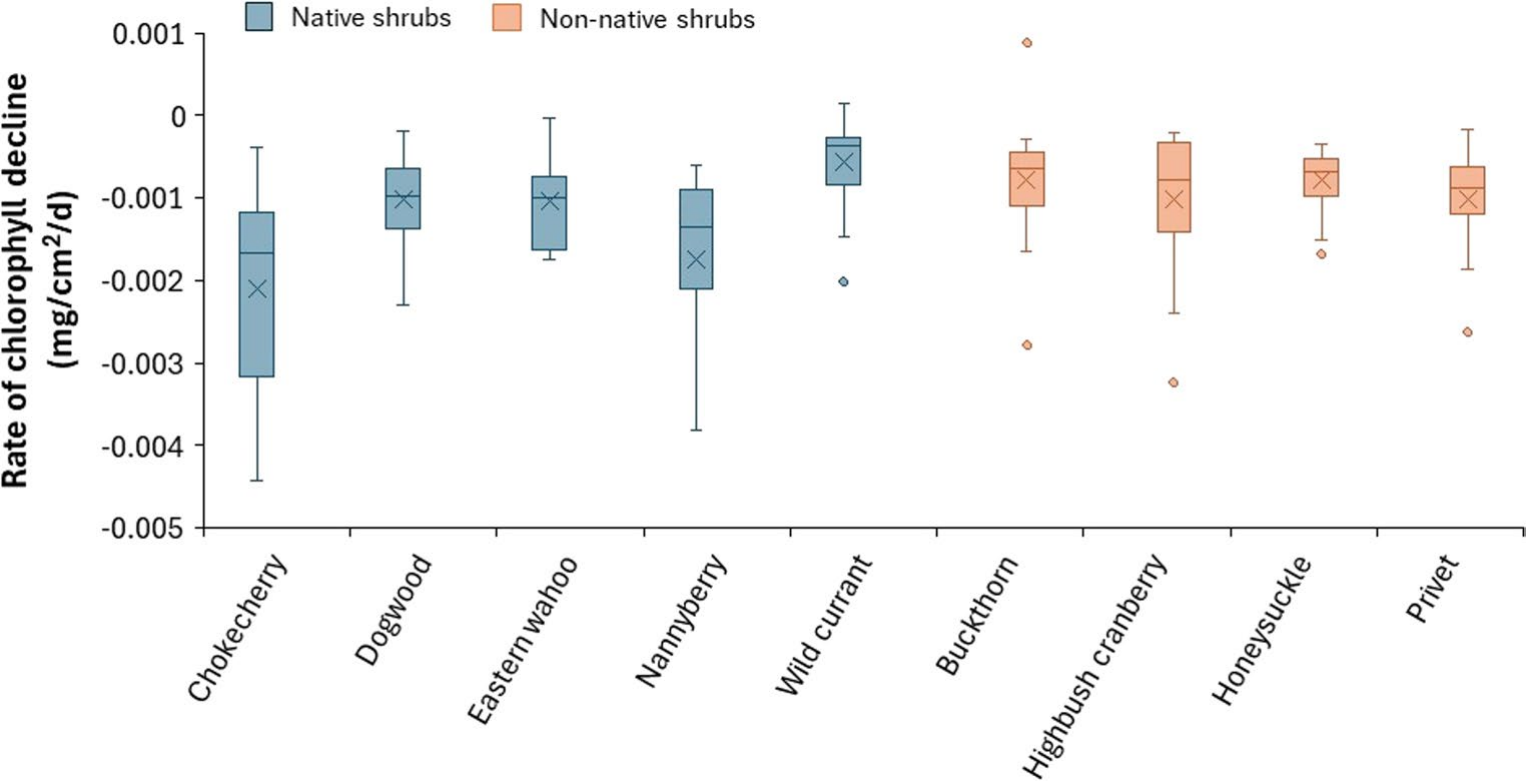
Average (2018, 2019, 2020, 2022, 2023 and 2024) rate of leaf chlorophyll decline (mg/cm^2^/d) for a range of 5 native temperate deciduous shrub species Chokecherry, Dogwood, Eastern wahoo, Nannyberry and Wild currant and 4 non-native shrub species Buckthorn, Highbush cranberry, Honeysuckle and Privet

The average (2018–2024) rate of leaf chlorophyll decline (mg/cm^2^/d) was also significantly (*p* < 0.004) faster (-0.0013 mg/cm^2^/d) in native shrubs compared to non-native shrubs (-0.0009 mg/cm^2^/d) (Fig. 4). However, this difference was only statistically significant in 2019 (Table 5).

**Table 5:** Pairwise comparison of the rate of leaf chlorophyll decline (mg/cm^2^/d average ± SE) in native (Nat, *N* = 89) and non-native (NonNat, *N* = 93) shrubs (2018–2024). P value indicates statistical significance

Year	Rate of chlorophyll decline (mg/cm^2^/d)		
	Nat	NonNat	*P* value
2018	-0.0010 ± 0.00022	-0.0010 ± 0.00021	0.702
2019	-0.0016 ± 0.00021	-0.0010 ± 0.00019	0.010
2020	-0.0013 ± 0.00024	-0.0010 ± 0.00022	0.431
2022	-0.0010 ± 0.00022	-0.0008 ± 0.00022	0.649
2023	-0.0013 ± 0.00023	-0.0007 ± 0.00025	0.090
2024	-0.0015 ± 0.00022	-0.0010 ± 0.00024	0.178

### Influence of temperature on autumn phenology in native and non-native shrubs 2018–2024

**Fig. 5:**
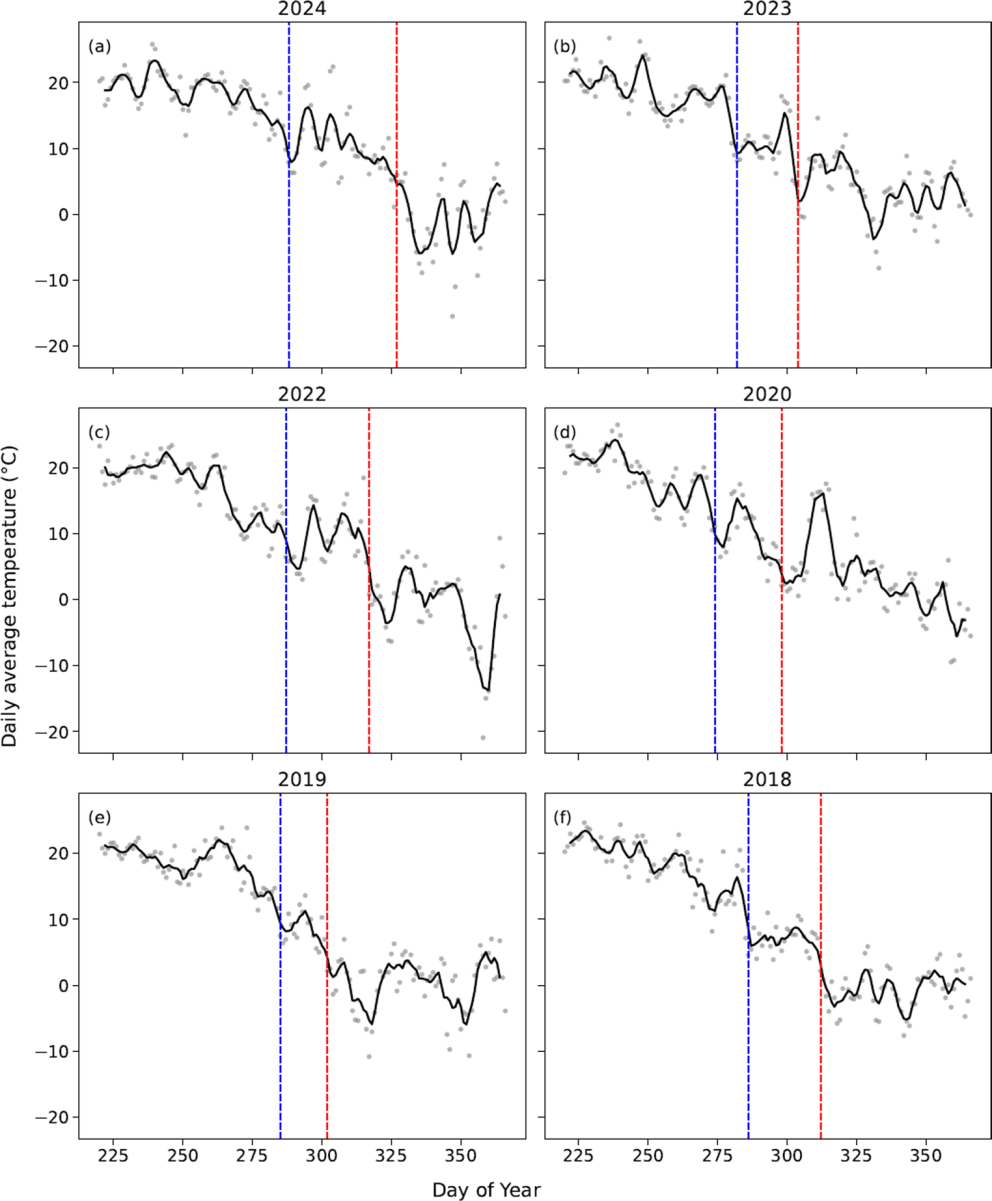
Average daily temperature (°C) from day of year (DOY) 220 to 365 for 2018, 2019, 2020, 2022, 2023 and 2024. Grey dots represent raw data, black line is the 5-day moving average, blue and red dotted lines represent the DOY of the first day in a three consecutive day period when daily average temperature was 10 °C–5 °C or below, respectively

The average daily temperature from DOY 220–365 together with the 5-year moving average shows a typical declining trend across years (Fig. 5). The average late summer (August; DOY 213–243) temperature (20.7 ± 0.38 °C) did not differ significantly (*p* = 0.102) across years (Table 6). The interannual timing of autumn cooling varied more for the 5 °C (29 days) than the 10 °C threshold (14 days) (Fig. 5). In 2024, an El Niño year, the timing of both thresholds, DOY 288 and 327 respectively was later than other years (DOYs 274–287; DOY 298–317) and the duration between them was also longer (39 days vs. 17–30 days) (Table 6; Fig. 5).

**Table 6:** Estimated onset (DOY) of sustained (3 consecutive days) periods below two temperature thresholds. Daily average August temperature (°C) ± standard error of the mean (SE)

Year	Temperature threshold		Daily average August temperature (°C) ± SE
	Below 10 °C	Below 5 °C	
2018	286	312	21.57 ± 0.387
2019	285	302	20.17 ± 0.296
2020	274	298	21.17 ± 0.443
2022	287	317	20.48 ± 0.337
2023	282	304	20.42 ± 0.390
2024	288	327	20.72 ± 0.421

Possibly due to the short duration of the time series, there were no statistically significant relationships detected between the three temperature parameters, average August temperature or start of autumn cooling (10 °C–5 °C thresholds) and the timing (DOY) and duration (days) of in situ leaf senescence or chlorophyll decline (Fig. S3).

## Discussion

### Use of SPAD meter in temperate deciduous forest research

Few studies have used the SPAD handheld chlorophyll meter in forest research and none to our knowledge have used it to characterize autumn senescence patterns across multiple temperate deciduous shrub species. Existing studies on woody plants have focused primarily on validating the effectiveness of SPAD measurements in estimating leaf chlorophyll content or physiological properties. For example, Coste et al. (2010) demonstrated strong relationships between SPAD values and measured leaf chlorophyll content of 8 species of tropical tree in French, Guiana while Uddling et al. (2007) showed that the relationship between SPAD readings and chlorophyll content in birch (wheat and potato) exhibited a curvilinear form. Beyond direct chlorophyll estimation, SPAD measurements have also been used to assess leaf health. Bonneville and Fyles (2006) linked SPAD values to foliar nitrogen status in trembling aspen (*Populus tremuloides* Michx.) in Quebec, Canada and Yan et al. (2025) found strong correlations between SPAD values, spectral vegetation indices and textural features in pear tree leaves in Xinjiang, China. Furthermore, Kato et al. (2024) used SPAD values to compare the effects of shading on leaf chlorophyll content of 8 evergreen deciduous species during autumn 2013 in Tokyo, Japan. Collectively, these studies demonstrate the reliability and versatility of SPAD measurements for estimating foliar chlorophyll and related traits in woody plants; however, despite this demonstrated utility, SPAD meters remain underused in forest phenology research (Coste et al. 2010), particularly for examining interspecific variation in foliar senescence patterns. 

### Differences in the timing and duration of autumn senescence and chlorophyll decline between native and non-native shrubs

At the whole plant level in situ observations over the 2018–2024 time period showed that native shrubs started showing visible signs of senescence consistently earlier than non-native species. On average, senescence began 2 weeks earlier in native shrubs which is consistent with previously published results from the same site albeit over a slightly different time period (2017–2020) (Donnelly et al. 2024b). An earlier start to senescence in native compared to non-native shrubs has also been reported previously in a range of locations and growth conditions (Harrington et al. 1989; Fridley et al. 2012; Chen and Matter 2017; Maynard-Bean et al. 2020; O’Connell and Savage 2020; Schuster et al. 2021). Furthermore, the duration of in situ senescence, a less reported pheno-metric, was roughly one week shorter in native shrubs than non-natives again consistent with previous studies (Donnelly et al. 2024b).

In contrast to in situ senescence observations, leaf chlorophyll decline is measured at the leaf level rather than the whole plant. On average foliar chlorophyll began to decline 3 weeks after visual observations, indicating a lag between initial leaf color change and measurable pigment loss. The onset of this decline was roughly 10 days earlier in native than non-native species but the duration of the decline was similarly one week shorter in native shrubs. These results are in agreement with reports from a common garden experiment (2008–2010) which found a 14 day delay in the timing (duration was not reported) of 50% chlorophyll breakdown between native and non-native temperate deciduous shrubs (Fridley 2012). Our findings suggest that early onset and shorter duration of senescence in native shrubs compared to non-native is observed at both the whole plant and leaf levels. Delayed senescence and prolonged chlorophyll retention by non-native shrubs may confer a competitive advantage if leaves remain photosynthetically active later in autumn. Even though non-native shrubs retain chlorophyll longer in autumn this does not mean the leaves are photosynthesizing (Donnelly et al. 2020; O’Connell and Savage 2020) as components of the photosynthetic apparatus may degrade before chlorophyll content shows signs of decline (Lichtenthaler 1987; Augspurger et al. 2005; Caplan et al. 2018). However, longer leaf retention may alter light levels and temperature on the forest floor which may alter future species composition by favoring recruitment of some species over others (Gorchov and Trisel 2003; Maynard-Bean and Kaye 2020).

Although differences in the onset of visual senescence were not statistically significant in all individual years, the direction of the response was consistent, and differences in chlorophyll decline were more robust across years. This suggests that chlorophyll dynamics may provide a more sensitive and mechanistic indicator of autumn phenology than visual assessments alone.

### Differences in the pattern and rate of chlorophyll decline between native and non-native plants

Prior to the general decline in leaf chlorophyll content, as indicated by the inflection point in Figs. 2, S1 & 1, native shrubs exhibited lower average leaf chlorophyll content (0.02–0.03 mg/cm^2^) compared to non-native species (0.04–0.05 mg/cm^2^) and a faster rate of decline. Together with the shorter duration of chlorophyll loss, these findings indicate a more rapid senescence process in native species suggesting a more efficient nutrient resorption strategy allowing nitrogen recovery prior to leaf abscission. In contrast, non-native shrubs maintained higher chlorophyll content later into the season and lost chlorophyll more gradually. These differing strategies may give native shrubs a competitive advantage as nitrogen recovery may be complete prior to the first hard freeze of the autumn season which prevents nutrient resorption (Estiarte and Peñuelas 2015; Schreiber et al. 2013). However, only in one year (2019) of this study did non-native shrubs still have at least some leaf chlorophyll (Fig. S2) after the first hard freeze (i.e. three consecutive days when daily average temperatures were below 0 °C) (Fig. 5) but if warmer autumns continue in future this pattern may change.

There was considerable interspecific and interannual variation in senescence patterns among both native and non-native shrubs. For example, Wild currant (Fig. S1w-ab) consistently exhibited lower leaf chlorophyll content and shorter duration of chlorophyll retention than other native species, whereas European privet (Fig. S2r-w) maintained the highest foliar chlorophyll levels and showed greater variability as chlorophyll declined among the non-native species. In addition, Buckthorn displayed a gradual decline in foliar chlorophyll content during autumn 2019 (Fig. S2b) but in 2020 (Fig. S2d) chlorophyll remained relatively high until late in the season when it declined rapidly. These findings suggest that senescence patterns are determined by a combination of species-specific traits and year-specific environmental conditions.

In general, increasing variability in leaf chlorophyll content as senescence progressed was observed in both shrub groups reflecting heterogeneity in pigment breakdown as leaves age as the season progresses. Chlorophyll degradation does not occur uniformly across the leaf. Senescence often begins near the leaf margins or tip and progresses inward, resulting in patchy chlorophyll loss. Leaf thinning, in deciduous broadleaf trees (Japan), has been observed during senescence which may account for variability in photosynthetic activity (Ito et al. 2006) and potentially chlorophyll content. Other factors that contribute to an uneven distribution of chlorophyll in a leaf include damage during the growing season from insect or a range of physical factors. All of these factors contribute to greater variability in chlorophyll content as leaves age.

### Influence of autumn temperature parameters on the timing and duration of senescence

Despite substantial interannual variability in autumn temperature and particularly for the timing of the 5 °C temperature threshold, no significant relationships were detected between temperature parameters (average August temperature, timing of 5 °C and 10 °C temperature thresholds) and the timing or duration of in situ senescence or chlorophyll decline, possibly reflecting the short duration of the time series. Whereas, warmer autumn temperatures have been widely reported to delay the timing of senescence across many temperate regions and measuring types, such as, in situ and satellite observations, these studies focus on trees and typically span a number of decades (Estrella and Menzel 2006; Gill et al. 2015; Liu et al. 2017; Zohner and Renner 2019; Kloos et al. 2024; Ji et al. 2025). Few studies include shrubs and those that do tend to be of short duration such as Fridley (2008–2010) and Panchen et al. 2015) (2012–2013) do not report significant relationships between autumn phenology (leaf fall) and temperature.

The El Niño year of 2024 was characterized by delayed autumn cooling and an extended period between the 10 °C and 5 °C thresholds. While this did not translate into statistically detectable shifts in senescence timing within the current dataset, such conditions may disproportionately favor non-native species that retain chlorophyll and leaves later into the season. Over longer time scales, repeated warm autumns could therefore amplify phenological differences between native and non-native shrubs, with implications for competitive interactions and ecosystem processes.

### Limitations of this study

SPAD-derived chlorophyll measurements provide detailed leaf-level information but do not capture spatial heterogeneity across entire canopies or shrubs. As a result, observed senescence patterns may not directly translate to canopy-scale optical properties or ecosystem-level carbon dynamics. Therefore, it may be useful in future to increase the number of leaves, species and sites for which SPAD readings are taken. This would certainly capture a wider range of variability in terms of species and geography, and we encourage researchers to conduct more studies like this one. The optimum number of each would depend on the objectives of the study and the availability of resources.

This analysis focused primarily on two air temperature metrics (average August temperature and autumn cooling thresholds) but this could be extended to explore a wider range of temperature parameters including average maximum and minimum temperatures, daytime and nighttime temperatures over different time period. In addition, other environmental factors known to influence autumn senescence—such as soil moisture, photoperiod interactions, drought stress, or extreme weather events (e.g., early frosts)—were not explicitly quantified and may contribute to unexplained variability, and should be considered in future work.

## Conclusion

The SPAD chlorophyll meter proved to be an effective and efficient tool for characterizing autumn senescence patterns across multiple temperate deciduous shrub species, enabling frequent, non-destructive estimates of foliar chlorophyll decline. Across the 2018–2024 study period, native and non-native shrubs exhibited differences in the timing, duration, and rate of senescence at both leaf and whole-plant scales. Native shrubs senesced earlier and lost chlorophyll more rapidly, completing senescence over a shorter period, whereas non-native shrubs maintained higher chlorophyll levels later into autumn and exhibited slower chlorophyll decline. These contrasting senescence strategies may have implications for late-season carbon uptake, nutrient resorption, and understory light conditions in temperate shrub communities. However, including a larger number of plants across a broader range of temperate deciduous forest sites would improve the robustness of these results and help determine whether differences in chlorophyll degradation between shrub groups are consistent. Interannual variation in autumn temperature was evident, but species origin emerged as a stronger determinant of senescence dynamics than temperature within the time frame of this study. Together, these results highlight the value of leaf-level chlorophyll measurements for resolving interspecific differences in autumn phenology and provide a foundation for future studies examining how shrub functional traits shape seasonal ecosystem processes.

## Supplementary Information

Supplementary Material 1. Table S1: List of data omitted from statistical analysis due to variation in rate of senescence across the leaf surface late in the senescence process resulting in anomalous SPAD value readings. Out of a total of +26,000 readings 31 were deemed unusable. Table S2: List of data omitted from statistical analysis due to negative chlorophyll content being returned due to very low SPAD reading. Number in parenthesis is the plant number. Out of a total of +26,000 readings 5 were deemed unusable. Table S3: Durbin–Watson statistics for residuals from piecewise linear regression models fitted to chlorophyll time series for each species–year combination. Values were used to assess first-order temporal autocorrelation in model residuals, with values near 2 indicating low autocorrelation, values <2 indicating positive autocorrelation, and values >2 indicating negative autocorrelation.

Supplementary Material 2. Figure S1: Piecewise linear regression for trends (day of year) in autumn leaf chlorophyll content (mg/cm2) for 5 native temperate deciduous shrub species (Eastern wahoo, Chokecherry, Pagoda dogwood, nannyberry and Wild currant) (a-ab) for 2018, 2019, 2020, 2022, 2023 and 2024. Note data for Eastern wahoo in 2018 and 2019 are missing. The red trend lines represent the fitted piecewise model segments and the blue dashed line the breakpoint for each fit. Figure S2: Piecewise linear regression for trends (day of year) in autumn leaf chlorophyll content (mg/cm2) for 4 non-native shrub species (Buckthorn, Highbush cranberry, Honeysuckle and European privet) (a-w) for 2018, 2019, 2020, 2022, 2023 and 2024. Note data for Highbush cranberry in 2023 are missing. The red trend lines represent the fitted piecewise model segments and the blue dashed line the breakpoint for each fit. Figure S3: Relationship between the start (a, b and c Day of year) and duration (d, e and f Days) of in situ senescence and chlorophyll decline in native and non-native shrubs and (a,d) the start of the first sustained 3-day period below 10°C (T10), (b,e) the start of the first sustained 3-day period below 5°C (T5) and (c,f) average August temperature (°C). Text within subplots shows the regression equation and the significance value (p). In situ Sen. Natives = the start (DOY) of in situ senescence in native shrubs; In situ Sen. Non-natives = the start (DOY) of in situ senescence in non-native shrubs; Chl. decline Natives = the start (DOY) of chlorophyll decline in native shrubs and Chl. decline Non-natives = the start (DOY) of chlorophyll decline in non-native shrubs. In situ Dur. Natives = the duration (Days) of in situ senescence in native shrubs; In situ Dur. Non-natives = the duration (Days) of in situ senescence in non-native shrubs; Chl. Dur. Natives = the duration (Days) of chlorophyll decline in native shrubs and Chl. Dur. Non-natives = the duration (Days) of chlorophyll decline in non-native shrubs.

## Data Availability

The phenology and chlorophyll data collected and the datasets used during the current study are available in the PANGAEA data repository (https://www.pangaea.de/) under the references cited Donnelly 2024a, b, 2025. The temperature data are available from the corresponding author on request as this data set is collected and kept locally.
