# Supplementary material for: Native shrubs senesce earlier and faster than non-native shrubs in a temperate deciduous woodland in south-eastern Wisconsin, USA
Source: Int J Biometeorol. 2026 Jun 22;70(7):194. doi: 10.1007/s00484-026-03252-y (PMC13287266; doi:10.1007/s00484-026-03252-y)
Supplement: Supplementary file 1 [file 484_2026_3252_MOESM1_ESM.odt]

Table S1 List of data omitted from statistical analysis due to variation in rate of senescence across the leaf surface late in the senescence process resulting in anomalous SPAD value readings. Out of a total of +26,000 readings 31 were deemed unusable.

| Year | Species | DOY | Justification |
| --- | --- | --- | --- |
| 2024 | Privet (2) | 274 & 277 | SPAD value increased from 27.6 (DOY 295) to 31.6 (DOY 298) |
| Honeysuckle (3) | 302 | SPAD value increased from 47.5 (DOY 270) to 51 and 56.3 (DOYs 274 & 277 respectively) |
| 2023 | Privet (4) | 303 | SPAD value increased from 37.7 (DOY 303) to 43.4 (DOY 306) due to two very low values (28 and 28.5) which were omitted from further analysis |
| Eastern wahoo (2) | 303 | SPAD value increased from 31 (DOY 303) to 40 (DOY 306) due to two very low values (16.6 and 17) which were omitted from further analysis |
| Honeysuckle (2) | 303 | SPAD value increased from 17.4 (DOY 300) to 24.9 (DOY 303) |
| Nannyberry (2) | 292 | SPAD value increased from 18.5 (DOY 289) to 25.6 (DOY 292) and back to 10 (DOY 296) |
| 2022 | Wild currant (2) | 262 | SPAD value increased from 18.8 (DOY 258) to 24.4 (DOY 262) |
| Wild currant (4) | 258 | SPAD value increased from 22.9 (DOY 255) to 27.2 (DOY 258) and back to 24.6 (DOY 262) |
| Buckthorn (5) | 290 | SPAD value increased from 19.3 (DOY 286) to 22.8 (DOY 290) and back to 17 (DOY 293) |
| Dogwood (3) | 290 | SPAD value (40) completely outside range for this species |
| Honeysuckle (3) | 293 | SPAD value increased from 17 (DOY 290) to 22.5 (DOY 293) and back to 8 (DOY 297) |
| 2020 | Eastern wahoo (2) | 307 | SPAD value increased from 17.1 (DOY 303) to 26.5 (DOY 307) and back to 10.5 (DOY 310) |
| 2019 | Privet (1) | 297 | SPAD value increased from 36.9 (DOY 295) to 44.2 (DOY 297) and back to 31.0 (DOY 299) |
| Chokecherry (2) | 255 | SPAD value increased from 25.1 (DOY 253) to 33.8 (DOY 255) and back to 24.4 (DOY 257) |
| Highbush cranberry | 267 | SPAD value increased from 18.9 (DOY 263) to 28.5 (DOY 267) and back to 16.5 (DOY 269) |
| Honeysuckle (1) | 281 & 283 | SPAD value increased from 33.7 (DOY 279) to 29.5 (DOY 281) and 38.7 (DOY 283) and back to 36.5 (DOY 285) |
| Honeysuckle (3) | 277 | SPAD value increased from 36.2 (DOY 273) to 40.4 (DOY 277) and back to 30.7 (DOY 279) |
| Honeysuckle (5) | 249 | SPAD value decreased from 39.6 (DOY 247) to 33.0 (DOY 249) and back to 43.0 (DOY 251) |
| Nannyberry (4) | 279 | SPAD value decreased from 16.2 (DOY 277) to 8.0 (DOY 279) and back to 15.2 (DOY 281) |
| 2018 | Highbush cranberry (1) | 264 | SPAD value increased from 25.8 (DOY 262) to 31.0 (DOY 264) and back to 25.7 (DOY 266) |
| Highbush cranberry (3) | 298 & 300 | SPAD value increased from 13.8 (DOY 296) to 24.1 (DOY 298) and 21.2 (DOY 300) and back to 12.8 (DOY 302) |
| Chokecherry (1) | 278 | SPAD value increased from 28.6 (DOY 276) to 33.6 (DOY 278) and back to 27.1 (DOY 280) |
| Buckthorn (1) | 278 | SPAD value decreased from 43.3 (DOY 276) to 33.6 (DOY 278) and back to 41.5 (DOY 280) |
| Buckthorn 2 | 264, 266, 290 &292 | SPAD value decreased from 36.1 (DOY 262) to 27.1 (DOY 264) and 24.1 (DOY 266) and back to 33.8 (DOY 268). SPAD value increased from 36.34.9 (DOY 288) to 42.0 (DOY 290) and 40.0 (DOY 292) and back to 32.0 (DOY 294). |
| Chokecherry (4) | 266 | SPAD value increased from 25.3 (DOY 264) to 31.5 (DOY 266) and back to 25.7 (DOY 268) |

Table S2 List of data omitted from statistical analysis due to negative chlorophyll content being returned due to very low SPAD reading. Number in parenthesis is the plant number. Out of a total of +26,000 readings 5 were deemed unusable.

| Species | Year | DOY | Replicate | SPAD | Chlorophyll |
| --- | --- | --- | --- | --- | --- |
| Lonicera morrowii Honeysuckle (3) | 2024 | 320 | 3 | 0.01 | -0.00003 |
| 2022 | 300 | 1 | 0 | -0.0001 |
| 2022 | 304 | 5 | 0 | -0.0001 |
| Rhamnus cathartica Buckthorn (2) | 2020 | 307 | 4 | 3.8 | -0.0005 |
| 2020 | 314 | 5 | 2.6 | -0.0017 |

Table S3 Durbin–Watson statistics for residuals from piecewise linear regression models fitted to chlorophyll time series for each species–year combination. Values were used to assess first-order temporal autocorrelation in model residuals, with values near 2 indicating low autocorrelation, values <2 indicating positive autocorrelation, and values >2 indicating negative autocorrelation.

| Species | Year | Durban-Watson value |
| --- | --- | --- |
| Buckthorn | 2018 | 1.62 |
| Buckthorn | 2019 | 1.89 |
| Buckthorn | 2020 | 1.53 |
| Buckthorn | 2022 | 1.94 |
| Buckthorn | 2023 | 2.23 |
| Buckthorn | 2024 | 1.73 |
| Eastern wahoo | 2020 | 2.18 |
| Eastern wahoo | 2022 | 2.21 |
| Eastern wahoo | 2023 | 3.03 |
| Eastern wahoo | 2024 | 2.15 |
| Chokecherry | 2018 | 2.56 |
| Chokecherry | 2019 | 0.95 |
| Chokecherry | 2020 | 1.89 |
| Chokecherry | 2022 | 2.16 |
| Chokecherry | 2023 | 3.33 |
| Chokecherry | 2024 | 3.01 |
| Dogwood | 2018 | 1.44 |
| Dogwood | 2019 | 2.12 |
| Dogwood | 2020 | 2.78 |
| Dogwood | 2022 | 1.77 |
| Dogwood | 2023 | 2.69 |
| Dogwood | 2024 | 2.23 |
| Highbush cranberry | 2018 | 1.15 |
| Highbush cranberry | 2019 | 1.11 |
| Highbush cranberry | 2020 | 2.28 |
| Highbush cranberry | 2022 | 1.99 |
| Highbush cranberry | 2024 | 0.99 |
| Honeysuckle | 2018 | 1.92 |
| Honeysuckle | 2019 | 1.74 |
| Honeysuckle | 2020 | 1.99 |
| Honeysuckle | 2022 | 2.55 |
| Honeysuckle | 2023 | 1.77 |
| Honeysuckle | 2024 | 2.54 |
| Nannberry | 2018 | 1.94 |
| Nannberry | 2019 | 1.60 |
| Nannberry | 2020 | 2.08 |
| Nannberry | 2022 | 2.06 |
| Nannberry | 2023 | 2.38 |
| Nannberry | 2024 | 2.87 |
| Privet | 2018 | 2.05 |
| Privet | 2019 | 1.21 |
| Privet | 2020 | 1.92 |
| Privet | 2022 | 1.13 |
| Privet | 2023 | 1.97 |
| Privet | 2024 | 2.36 |
| Wild currant | 2018 | 2.60 |
| Wild currant | 2019 | 2.21 |
| Wild currant | 2020 | 1.92 |
| Wild currant | 2022 | 1.72 |
| Wild currant | 2023 | 1.16 |
| Wild currant | 2024 | 3.29 |
