# Supplementary material for: Native shrubs senesce earlier and faster than non-native shrubs in a temperate deciduous woodland in south-eastern Wisconsin, USA
Source: Int J Biometeorol. 2026 Jun 22;70(7):194. doi: 10.1007/s00484-026-03252-y (PMC13287266; doi:10.1007/s00484-026-03252-y)
Supplement: Supplementary file 2 [file 484_2026_3252_MOESM2_ESM.odt]

Figure S1 Piecewise linear regression for trends (day of year) in autumn leaf chlorophyll content (mg/cm2) for 5 native temperate deciduous shrub species (Eastern wahoo, Chokecherry, Pagoda dogwood, nannyberry and Wild currant) (a-ab) for 2018, 2019, 2020, 2022, 2023 and 2024. Note data for Eastern wahoo in 2018 and 2019 are missing. The red trend lines represent the fitted piecewise model segments and the blue dashed line the breakpoint for each fit.

Figure S2 Piecewise linear regression for trends (day of year) in autumn leaf chlorophyll content (mg/cm2) for 4 non-native shrub species (Buckthorn, Highbush cranberry, Honeysuckle and European privet) (a-w) for 2018, 2019, 2020, 2022, 2023 and 2024. Note data for Highbush cranberry in 2023 are missing. The red trend lines represent the fitted piecewise model segments and the blue dashed line the breakpoint for each fit.

Figure S3 Relationship between the start (a, b and c Day of year) and duration (d, e and f Days) of in situ senescence and chlorophyll decline in native and non-native shrubs and (a,d) the start of the first sustained 3-day period below 10°C (T10), (b,e) the start of the first sustained 3-day period below 5°C (T5) and (c,f) average August temperature (°C). Text within subplots shows the regression equation and the significance value (p). In situ Sen. Natives  = the start (DOY) of in situ senescence in native shrubs; In situ Sen. Non-natives = the start (DOY) of in situ senescence in non-native shrubs; Chl. decline Natives = the start (DOY) of chlorophyll decline in native shrubs and Chl. decline Non-natives = the start (DOY) of chlorophyll decline in non-native shrubs. In situ Dur. Natives  = the duration (Days) of in situ senescence in native shrubs; In situ Dur. Non-natives = the duration (Days) of in situ senescence in non-native shrubs; Chl. Dur. Natives = the duration (Days) of chlorophyll decline in native shrubs and Chl. Dur. Non-natives = the duration (Days) of chlorophyll decline in non-native shrubs.
